# Mesenteric Lymphatic B Cells Migrate to the Intestine and Aggravate DSS-Induced Colitis via the CXCR5–CXCL13 Axis

**DOI:** 10.3390/biology13050322

**Published:** 2024-05-06

**Authors:** Yu Zhang, Zhe Wu, Qinghe Zhao, Yaming Liu, Qing Huang, Menglei Zhang, Shuolei Li, Di Wang, Na Li, Yujing Chi, Yulan Liu

**Affiliations:** 1 Department of Gastroenterology, Peking University People’s Hospital, Beijing 100044, China; zhangyu_zxy@bjmu.edu.cn (Y.Z.); samplezhewu@bjmu.edu.cn (Z.W.); 2211210258@stu.pku.edu.cn (Q.Z.); 2 Clinical Center of Immune-Mediated Digestive Diseases, Peking University People’s Hospital, Beijing 100044, China; 3 Department of Central Laboratory and Institute of Clinical Molecular Biology, Peking University People’s Hospital, Beijing 100044, China; ellen_wd@sina.com (D.W.); yingshw@163.com (N.L.); 4 Department of Gastroenterology and Hepatology, Xiamen University Zhongshan Hospital, Xiamen 361001, China; yaming0856@gmail.com; 5 Department of Gastroenterology, Beijing Tsinghua Changgung Hospital, School of Clinical Medicine, Tsinghua University, Beijing 102218, China; huangqingevery@163.com; 6 Department of Animal Laboratory, Peking University People’s Hospital, Beijing 100044, China; zhangmenglei@pkuph.edu.cn (M.Z.); lishuolei@pkuph.edu.cn (S.L.)

**Keywords:** mesenteric lymphocytic B cells, inflammatory bowel disease, CXCR5-CXCL13 axis, T cells

## Abstract

**Simple Summary:**

Inflammatory bowel disease (IBD) is characterized by mucous bloody stools, abdominal pain, and diarrhea. Once diagnosed with IBD, the patients often rely on medication for several years due to its incurability, and some may require surgical treatments. The pathogenesis of IBD is still unrevealed. It is believed to result from excessive immune and inflammatory responses to environmental factors in genetically susceptible individuals. The successful use of various biological agents (infliximab, adalimumab, and vitolizumab) has also confirmed the role of immune response in IBD. Mesenteric lymphatics penetrate the intestinal wall and form an immune complex with the intestine. Therefore, we aim to explore the role of mesenteric lymphatic immunity in IBD. We observed that the proportion of mesenteric lymphatic B (MLB) cells increased in IBD rats. The adoptive transfer of MLB cells aggravated IBD in the receipt rats, accompanied by changes in intestinal T cell number and function. Additionally, we identified the CXCR5-CXCL13 axis as being involved in inducting the migration of MLB cells to the intestine, indicating that MLB cells play important roles in IBD.

**Abstract:**

The pathogenesis of inflammatory bowel disease (IBD) is still unknown. Mesenteric lymphatics (MLs), which are closely related to the intestine in both anatomy and physiology, have been suggested to be involved in IBD. In the present study, we aim to investigate the effects of ML immune cells on IBD and explore the potential associated mechanisms. Acute colitis was induced in rats using dextran sulfate sodium salt (DSS). Mesenteric lymphangiogenesis, ML stenosis, and dilation were observed, with an increased proportion of MLB cells in DSS-induced colitis rats. The adoptive transfer of B cells isolated from ML (MLB) was employed to investigate their effects on colitis. MLB cells derived from DSS-induced colitis rats exhibited a higher propensity to migrate to the intestine. The proportion of colonic T cells was altered, along with the aggravated colitis induced by the adoptive transfer of MLB cells derived from DSS-induced colitis rats. RNA sequencing revealed increased *Cxcr5* expression in MLB cells from colitis rats, while real-time PCR indicated an upregulation of its ligand *Cxcl13* in the colon of colitis rats. These findings suggest that MLB cells may migrate to the intestine and aggravate colitis. In summary, colonic T cells respond to MLB cells from colitis rats, and MLB cells aggravate DSS-induced colitis via the CXCR5–CXCL13 axis.

## 1. Introduction

The incidence of inflammatory bowel disease (IBD), including ulcerative colitis (UC) and Crohn’s disease (CD), is increasing worldwide. It is widely accepted that IBD results from excessive immune and inflammatory responses to environmental factors in genetically susceptible individuals [1,2]; however, the pathogenesis of IBD still remains unknown. Interstitial fluids and immune cells from the intestinal tract drain into mesenteric lymphatics, forming lymph, which is transported through the mesenteric lymph nodes (MLNs) and eventually back into the circulating blood [3,4]. Emerging evidence has demonstrated the potential role of mesenteric lymphatics in the pathogenesis of IBD. Alterations in the morphology and dysfunction of mesenteric lymphatics are often observed in IBD patients [5,6], as well as in colitis mice [7]. Moreover, the mesenteric lymphatic density is associated with the development of IBD [5]. In animal studies, mice with structural (dilated torturous lymphatic vessels) and functional (greater sub-mucosal edema and higher immune cell burden) alterations are more susceptible to colitis and the development of aggravated intestinal inflammation [8,9]. Under tumor necrosis factor (TNF) stimulation, the impaired function of mesenteric lymphatic drainage of lymph to the MLN may be involved in the pathogenesis of IBD [10]. Additionally, it has been found in IBD models that drugs targeting the release from mesenteric lymphatic vessels and promoting lymphatic drainage can alleviate intestinal inflammation [11].

The anatomical and physiological features of mesenteric lymphatics indicate their immune function. Lymph node B cells have been found to orchestrate the expansion of the lymphatic network, as well as aiding migration of dendritic cells (DCs) from the periphery [12]. In the mesenteric tissue of IBD patients, GJ Randolph et al. observed that B cell-rich aggregates impinged on lymphatics entering and exiting lymph nodes, and B cells had invaded the lymphatic vessel wall in creeping fat [13]. These findings reveal that B cells may be involved in lymphatic remodeling and affect other immune cells. Unfortunately, the immune mechanisms of mesenteric lymphatics in IBD are poorly understood. Here, we aim to explore the role of mesenteric lymphatic B (MLB) cells in dextran sulfate sodium salt (DSS)-induced colitis and explore the potential associated mechanisms.

In this study, we demonstrate that the number of mesenteric lymphatic B cells were increased in colitis using flow cytometry. Lymphatic structure was evaluated using immunohistochemistry. Mesenteric lymphatic B cells could migrate to the intestine, as observed using intravital imaging. Mesenteric lymphatic B cells aggravate DSS-induced colitis, as evidenced by adoptive transfer, which may also be related to colonic T cells. The CXCR5–CXCL13 axis was identified to induce the migration of mesenteric lymphatic B cells through RNA sequencing, real-time PCR, and cell co-culture.

## 2. Materials and Methods

### 2.1. Rats and Induction of Colitis

The animal experiments involved in this study were approved by the Institutional Animal Care and Use Committee of Peking University People’s Hospital (document numbers: 2019PHE006, 2020PHE025, 2022PHE143). Sprague Dawley (SD) wild-type male rats (6 to 8 weeks old) were provided by Beijing Huafukang Biotechnology Co., Ltd., and were raised in the specific pathogen-free facility of the Animal Experiment Center of Peking University People’s Hospital. Six rats were housed in a cage in a conditioned environment with a 12 h light and dark cycle, a temperature of 18 to 23 °C, and a humidity of 40 to 60%. Acute colitis was induced by 5% dextran sulfate sodium (DSS, 36,000–50,000 MW, MP Biomedicals) for 7 days. There were 6 rats in each cage and 5% DSS drinking water was refreshed every day. All rats used were monitored for body weight, diarrhea, and hematochezia for assessment of colitis symptoms.

### 2.2. Collecting Mesenteric Lymph

First, 5% heparin was used to flush the lymphatic pipette, 1.5 mL EP tube, and 25 G needle to ensure that they were patent. The rats were anesthetized with isoflurane inhalation, then fixed on a clean surgical drape on a heating plate (37 ℃). The middle part of the abdomen was shaved and disinfected three times with iodophor and 70% ethanol. Then, we opened the middle abdomen with a 2 to 4 cm incision, opened the remaining layers of the abdominal muscle wall from 4 to 5 mm lateral to the midline to the right flank with a small pair of surgical scissors, and used 2–3 pieces of sterile gauze soaked in sterile saline to fasten the small intestine under the left abdominal muscle wall. Then, we found the white pipe with a diameter of 0.5 to 1 mm perpendicular to the right kidney, close to and parallel with the mesenteric artery. Mesenteric lymphatic vessels were bluntly separated from connective tissue and fat, a 25 G needle was used to make a small hole in the lymphatic vessel, and a lymphatic suction tube was placed at the lymphatic vessel hole to collect lymph fluid. We injected 50 μL of Carbon Nanoparticle Suspension (H20073246, Chongqing Lummy Pharmaceutical Co., Ltd., China) into Peyer’s patch in the gut.

### 2.3. Magnetic Microbead-Based Cell Sorting

Single-cell suspensions of mesenteric lymph lymphocytes were prepared under sterile conditions. The desired cells were labeled with CD45R antigen (CD45RA, Miltenyi, 130-090-494). The labeled positive fraction (CD45RA cells) was obtained with the OctoMACS Separator (Miltenyi, 130-042-109) and MACS Multi-Stand (Miltenyi, 130-042-303).

### 2.4. Mesenteric Lymphatic B Cells Adoptive Transfer and Intravital Imagination

A total of 5 × 10^6^ mesenteric lymphatic B cells were labeled with DiR (Invitrogen, D12731) and adoptive transfer was conducted through the tail vein. The cells were observed using an IVIS Spectrum (PerkinElmer) at 2 h, 18 h, 24 h, and 48 h after transfer. In particular, 5 × 10^6^ mesenteric lymphatic B cells from NC rats or DSS rats were adoptively transferred through the tail vein 3 times, on the 2nd, 4th, and 6th day during the onset of acute colitis. 

### 2.5. Hematoxylin and Eosin (H&E) Staining, and Immunohistochemistry (IHC)

The distal colon tissues of rats were fixed in 4% paraformaldehyde and embedded in paraffin. For hematoxylin and eosin (H&E) staining, tissue sections were stained with hematoxylin and eosin. For immunohistochemistry (IHC), paraffin sections were dewaxed, antigen recovered, endogenous peroxidase blocked, blocked with goat serum, and incubated with primary antibody (podoplanin antibody, 1: 100, Santa, sc-166906) in a humidified box at 4 °C overnight. The paraffin sections were then washed three times with PBS and incubated with the secondary antibody (goat anti-mouse/rabbit IgG polymer, Zhongshan Jinqiao, PV-8000). A microscope (ZEISS, Axio Scope A1) was used to observe and collect images.

### 2.6. Bulk RNA Sequencing and Bioinformatic Analysis

RNA was extracted with TRIzol (ThermoFisher, 15596018) from mesenteric lymphatic B cells. RNA degradation and contamination was monitored on 1% agarose gels, RNA purity was checked using a NanoPhotometer spectrophotometer (IMPLEN, CA, USA), and RNA integrity was assessed using the RNA Nano 6000 Assay Kit of the Bioanalyzer 2100 system (Agilent Technologies, CA, USA). Sequencing libraries were generated using an NEBNextUltraTM RNA Library Prep Kit for Illumina (NEB, USA), following the manufacturer’s recommendations. Then, the index codes were added to attribute sequences to each sample, and the clustering of the index-coded sample was performed on a cBot Cluster Generation System using TruSeq PE Cluster Kit v3-cBot-HS (Illumina), according to the manufacturer’s instructions. The library was sequenced using an Illumina Novaseq 6000. The fastq-formatted raw data were processed using in-house perl scripts. The DESeq2 R package (1.16.1), the edgeR R package (3.18.1), and KEGG enrichment analysis were used for differential expression analysis and statistical enrichment of differential expression genes in KEGG pathways. The definition of B cell signature referred to Boros [14] and Kovalova [15].

### 2.7. Real-Time Quantitative Polymerase Chain Reaction (RT-qPCR)

RNA extraction was performed using TRIzol Reagent (ThermoFisher, 15596018), and total RNA was reverse transcribed using a reverse transcriptase kit (ThermoFisher, K1622). The primer sequences were as follows: *Cxcl13*: forward primer: 5′-CTGCCTCCCTCCAGGCCACGG-3′, reverse primer: 5′-TGGGGCAGCCATTCCC-AGGGCGTA-3′. *Cxcr5*: forward primer: 5′-ACTCCCCGATATCGCTAGACA -3′, reverse primer: 5′-TTGATCTTGTGCAGGGCGAT -3′. *Il6:* forward primer: 5′-AGAGACTTCCAGCCAGTTGC-3′, reverse primer: 5′-AGTCTCCTCTCCGGACTTGT-3′. *Cd40:* forward primer: 5′-GTTGGGACCCCTGTGATCTG-3′, reverse primer: 5′-ACTGTCCTAGATGGACCGCT-3′. RT-qPCR was performed with appropriate cDNA and primers in SYBR Green Master Mix (ThermoFisher, A25742) on the StepOne Plus Real-Time PCR System (ThermoFisher, 4376592).

### 2.8. Immunocyte Isolation and Flow Cytometry

The immune cells of colonic lamina propria were isolated as previously described. Mesenteric lymph lymphocytes were isolated from mesenteric lymph through centrifuging for 5 min at 500× *g* and 4 °C. The single-cell suspensions were incubated at 37 °C for 5 h with Cell Stimulation Cocktail plus protein transport inhibitors (ThermoFisher, 00-4975-03) and blocked with CD32 (BD Bioscience, 550271). For live/dead staining, cell suspensions were stained with Fixable Viability Dyes (eBioscience, 65-0866-14,1 µL/ million cells). For cell surface marker staining, cell suspensions were stained with CD45 (Biologend, 202214, Clone: OX-1, 0.25 µg/million cells), B220 (BD Biosciences, 743590, Clone: HIS24, 0.25 µg/million cells), CD3 (Biolegend, 201403, Clone: 1F4, 0.25 µg/million cells), CD4 (Biolegend, 201520, Clone: W3/25, 0.25 µg/million cells), and CD8 (eBioscience, 56-0084-82, Clone: OX8, 1 µg/million cells) at 4 °C for 30 min. For intracellular cytokine staining, cells were stained with IL-10 (BD Bioscience, 555088, Clone: A5-4, 0.5 µg/million cells), IFN-*γ* (Biolegend, 507806, Clone: DB-1, 5 µL/million cells), IL-4 (Invitrogen, 50-7045-82, Clone: OX81, 0.5 µg/million cells), and IL-17 (Invitrogen, 48-7177-82, Clone: eBio17B7, 0.25 µg/million cells) after fixation and permeabilization using Fixation Buffer (Biolegend, 420801) and intracellular staining (Biolegend, 421002). The gating strategy is presented in Figure S1. The unstained and the single-color control samples were utilized to calculate compensation [16], and Fluorescence Minus One (FMO) controls were employed to establish the cut-off values of cytokines for negative and positive determinations. The level of expression of the B220 clone is an indicator of both maturational stages in the B cell lineage and functionally distinct B cell subsets. The B cells were defined as shown in Figure S1.

### 2.9. Knockdown of Cxcl13 Expression in IEC-6 Cells

IEC-6 (Intestinal Epithelioid Cell line No. 6) cells are a cell line derived from rat intestinal epithelial cells. Rat-specific small interfering RNA (siRNA) was used to knock down the mRNA expression of *Cxcl13*. The sequences of the three siRNAs (Beijing Tsingke Biotech Co., Ltd.) are as follows: siCXCL13-1: sense: CCGAAGAAGUAUUACUUCA, antisense: UGAAGUAAUACUUCUUCGG. siCXCL13-2: sense: GCUAUAUGUGUGAAU-CCUA, antisense: UAGGAUUCACACAUAUAGC; siCXCL13-3: sense: CGACCUUUAU-CAAUCUAAU; antisense: AUUAGAUUGAUAAAGGUCG. IEC-6 cells were cultured in a 6-well plate with DMEM complete medium (10% FBS, 100 U/mL penicillin–streptomycin solution) and placed in a 37 °C, 5% CO_2_ incubator. Once the cells reached 60%~70% confluence, control or siCXCL13 (50 nmol/L) and transfection reagent (5 μL, Biodragon, KX0110049) in 5% glucose solution (100 μL) was mixed and incubated at room temperature for 20 min. Then, the mixture was added to IEC-6 cells in serum-free and antibiotic-free culture medium. After 24 h of treatment, RNA was extracted for real-time PCR to detect the efficiency of the three siRNAs. siCXCL13-2 was finally selected for formal experiments.

### 2.10. Co-Culture of IEC-6 and Mesenteric Lymphatic B Cells

IEC-6 cells were plated in a 24-well confocal dish (Cellvis, P24-1.5H-N) and treated with siCXCL13-2 for 24 h. Subsequently, the Transwell chamber (Corning, 3415) was placed in the confocal dish, and the sorted mesenteric lymphatic B cells from normal control rats and rats with DSS-induced colitis were inoculated into the upper chamber for co-culture. The groups were designated as follows: mesenteric lymphatic B cells derived from normal control rat co-culture with IEC-6 cells (NC MLB+IEC-6), mesenteric lymphatic B cells derived from normal control rat co-culture with IEC-6-knocked-down CXCL13 cells (NC MLB+IEC-6-CXCL13 KD), mesenteric lymphatic B cells derived from DSS-induced colitis rat co-culture with IEC-6 cells (DSS MLB+IEC-6), mesenteric lymphatic B cells derived from DSS-induced colitis rat co-culture with IEC-6-knocked-down CXCL13 cells (DSS MLB+IEC-6-CXCL13 KD). Each group was replicated in triplicate. Over the 12 to 24 h of co-culture conditions, the Multi-mode Intelligent Living Cells Imaging Analysis System (Leica) was used for live cell imaging tracking. MLB cells were small, round, and translucent, much smaller than IEC-6 cells, and were evenly distributed in the field of view. After 24 h of co-culture, four fields of 200× magnification in each well were randomly selected. The number of migrating B cells into the lower chamber in each field was then counted, and statistical analysis was conducted.

### 2.11. Statistical Analysis

All results were expressed as mean ± SEM. Statistical significance between two groups, assuming Gaussian distribution, was evaluated using Student’s t-test, and significance between two groups without such assumption was assessed using Mann–Whitney tests. Comparisons among multiple groups were assessed by one-way analysis of variance (ANOVA) and Fisher’s LSD test. All data statistical analysis was conducted by GraphPad Prism 9.5 (GraphPad Software, La Jolla, CA, USA).

## 3. Results

### 3.1. DSS-Induced Colitis of Rats

**Figure 1:**
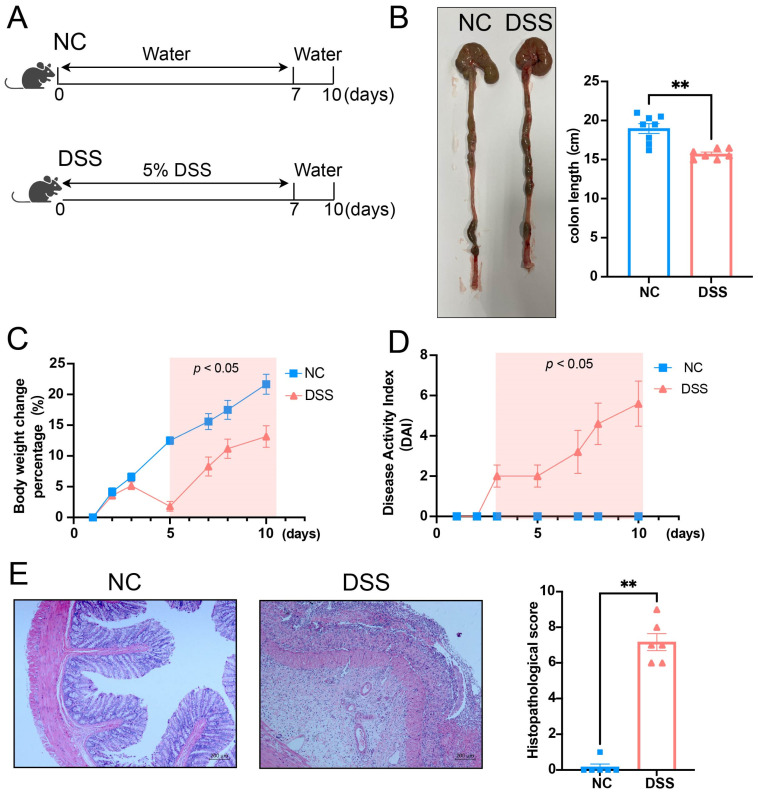
Model of DSS-induced acute colitis in rats. (**A**) The DSS group was induced by free drinking 5% DSS for 7 days, followed by water for 3 days. The NC group was treated with free drinking water for 10 days. (**B**) Representative colon-length images for the NC and DSS groups are shown in the left panel, and statistical analysis is shown in the right panel (*p* = 0.0011). (**C**) The body weight change percentage between NC and DSS groups. (**D**) The disease activity index (DAI) between NC and DSS groups. (**E**) Representative histopathological images of the NC and DSS groups are shown in the left panel, and the statistical analysis of the two groups is shown in the right panel (*p* = 0.0022). NC, normal control; DSS, DSS-induced colitis; *n* = 6; Mann–Whitney test; ** *p* < 0.01 with NC rats.

The protocol for inducing acute colitis in rats using DSS is shown in Figure 1A. Compared to the normal control (NC) rats, the DSS-induced colitis rats exhibited a shorter colon length (Figure 1B), lower body weight (Figure 1C), and a higher disease activity index (Figure 1D). Additionally, the histopathological score of the colon in DSS-induced colitis rats was significantly enhanced compared to the NC rats (Figure 1E). 

**Figure 2:**
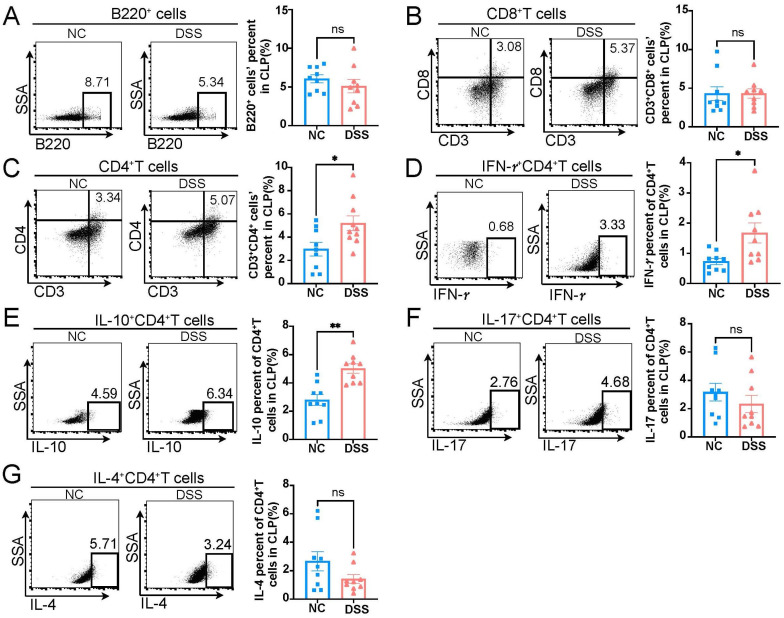
The changes in adaptive immune cells in colonic lamina propria of DSS-induced colitis rats. (**A**–**G**) Representative flow cytometry images of adaptive immune cells in the colonic lamina propria (CLP) of NC and DSS rats are shown in the left panel, and statistical analysis between two groups is shown in the right panel. NC, normal control; DSS, DSS-induced colitis; B cells, A, *p* = 0.3281; CD8^+^T cells, B, *p* = 0.5457; CD4^+^T cells, C, *p* = 0.0435; IFN-*γ*^+^CD4^+^ T cells, D, *p* = 0.0188; IL-10^+^CD4^+^ T cells, E, *p* = 0.0017; IL-17^+^CD4^+^ T cells, F, *p* = 0.3401; IL-4^+^CD4^+^ T cells, G, *p* = 0.2581; CLP, colonic lamina propria; *n* = 9; Mann–Whitney test; * *p* < 0.05 with NC rats; ns, no significant difference.

The colonic immune response actively contributes to colonic inflammation in IBD; thus, B cells and T cells in the colon were detected. When compared to the adaptive immune cells in colonic lamina propria (CLP) of the NC group, the CLP of the DSS group exhibited similar proportions of B220^+^ B cells (Figure 2A) and CD3^+^CD8^+^ T cells (Figure 2B), and higher CD3^+^CD4^+^ T cells (Figure 2C). Additionally, the proportions of IFN-γ^+^CD4^+^ T (Th1) cells and IL-10^+^CD4^+^ T cells were increased in the CLP of DSS-induced colitis rats, while the proportions of IL-17^+^CD4^+^ T (Th17) cells and IL-4^+^CD4^+^ T (Th2) cells showed no significant difference between the two groups (Figure 2D–G).

### 3.2. Morphology and Immune Cell Profile Changes in The Mesenteric Lymphatics of DSS-Induced Colitis Rats

**Figure 3:**
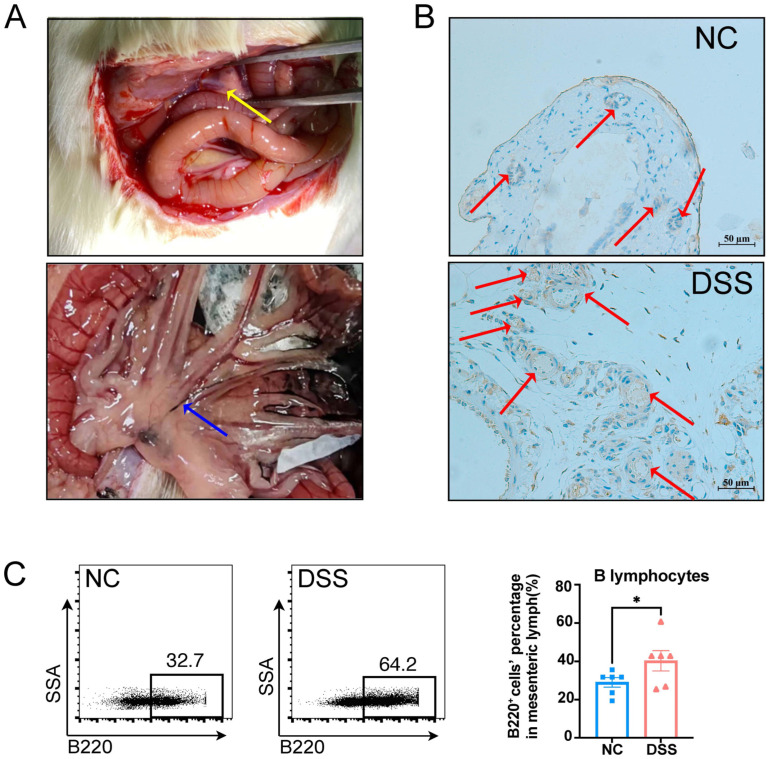
The changes in morphology and immune cell profile in the mesenteric lymph of DSS-induced colitis rats. (**A**) The mesenteric lymphatics in rats are depicted (the yellow arrow indicates primary mesenteric lymphatics, while the blue arrow indicates mesenteric lymphatics labeled with nanocarbon in rats). (**B**) Representative immunohistochemical images of mesenteric lymphatics (podoplanin, lymphatic vessels endothelial cell maker) in both NC and DSS rats. The red arrows indicate the lymphatic vessels. (**C**) Representative flow cytometry images of mesenteric lymphatic B lymphocytes are shown in the left panel, while the statistical analysis between the two groups is shown in the right panel (*p* = 0.0415). NC, normal control; DSS, DSS-induced colitis. *n* = 6; Mann–Whitney test; * *p* < 0.05 with NC rats.

To explore the alteration in mesenteric lymphatics and mesenteric lymphatic B lymphocytes in DSS-induced colitis rats, we isolated mesenteric lymphatics and mesenteric lymph to perform immunohistochemistry and flow cytometry analysis. As shown in Figure 3A, the mesenteric lymphatics was an opaque white vessel approximately 0.5 to 1 mm in diameter with pulsation, which turned black after nanocarbon injection from Peyer’s patch. Podoplaninisa is a specific lymphatic endothelial marker that is not expressed in the vascular endothelium [17]. Compared to the NC rats, the expression of podoplanin was increased in the mesenteric lymphatics of DSS-induced colitis rats (Figure 3B). Narrowing and relative expansion of mesenteric lymphatics were observed in DSS-induced colitis rats but not in NC rats (Figure 3B). Furthermore, B220^+^ B lymphocytes in mesenteric lymph were significantly increased in DSS-induced colitis rats, compared to those in NC rats. These findings indicate that mesenteric lymphatic B lymphocytes might be involved in the mechanism of colitis.

### 3.3. Adoptive Transfer Lymphatic B Cells from DSS-Induced Colitis Rats Aggravates DSS-Induced Colitis

**Figure 4:**
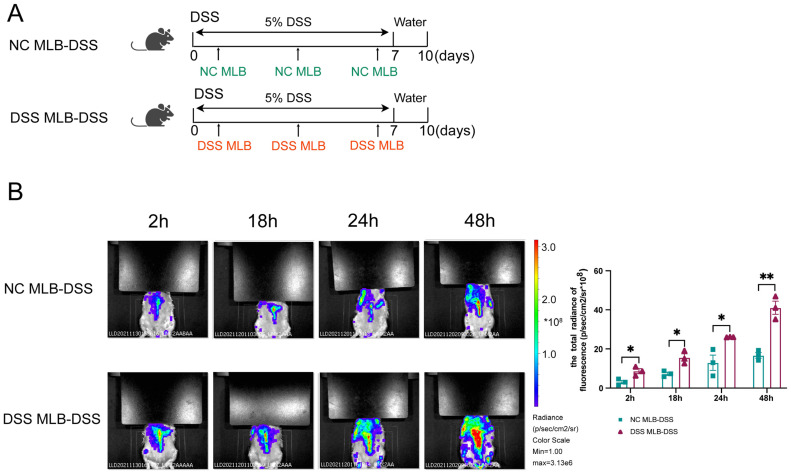
MLB cells migrate to the colon in DSS-induced colitis rats. (**A**) 5 × 10^6^ NC MLB or DSS MLB labeled with DiR were adoptively transferred into DSS-induced colitis rats three times during 5% DSS treatment. (**B**) Representative fluorescence images of the NC MLB-DSS group and the DSS MLB-DSS group are presented in the left panel, and the statistical analysis between the two groups is shown in the right panel at 2 h (hour) (*p* = 0.0112), 18 h (*p* = 0.0213), 24 h (*p* = 0.0144), and 48 h (*p* = 0.0013) after the adoption of NC MLB or DSS MLB, respectively. *n* = 3–6, Mann–Whitney test; NC MLB, mesenteric lymphatic B cells derived from the normal control rat; DSS MLB, mesenteric lymphatic B cells derived from DSS-induced colitis rat; NC MLB-DSS, the DSS-induced colitis rats receiving NC MLB; DSS MLB-DSS, the DSS-induced colitis rats receiving DSS MLB; * *p* < 0.05, ** *p* < 0.01 with NC rats; h, hour.

The CD45RA isoform is specific to the B cell lineage and is not expressed on thymocytes or any peripheral T cell subset. To investigate the role of mesenteric lymphatic B cells in colitis, we sorted mesenteric lymphatic B (MLB) cells using positive selection with rat CD45RA microbeads. The purity and viability of microbead sorting were over 95%. Then, we transferred them to the DSS-induced colitis rats through tail vein injection. The transfer protocol is depicted in Figure 4A. Intravital imaging was utilized to explore the migration of MLB in vivo. The isolated MLB cells derived from different groups were labeled with DiR. As shown in Figure 4B, DSS-induced colitis rats receiving MLB cells from DSS-induced colitis (DSS MLB-DSS) rats exhibited increased fluorescence density in the intestine at various time points, compared to those receiving MLB cells from NC (NC MLB-DSS) rats. This suggests that MLB cells derived from DSS-induced colitis rats are more prone to migrating to the intestines. 

**Figure 5:**
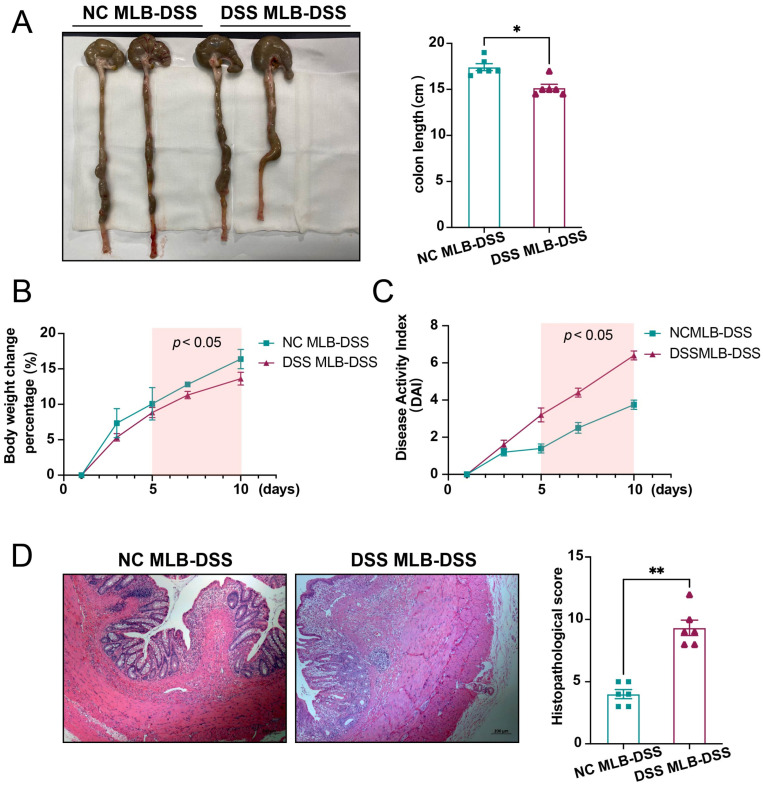
MLB cells derived from DSS rats aggravate DSS-induced colitis. (**A**) Representative colon-length images of the NC MLB-DSS or DSS MLB-DSS are shown in the left panel, and the statistical analysis between the two groups is shown in the right panel (*p* = 0.0397). (**B**) The body weight change percentage in the NC MLB-DSS and DSS MLB-DSS groups (*p* < 0.05). (**C**) The disease activity index (DAI) in the NC MLB-DSS and DSS MLB-DSS groups (*p* < 0.05). (**D**) Representative histopathological images of the NC MLB-DSS and DSS MLB-DSS groups are shown in the left panel, while the statistical analysis between the two groups is displayed in the right panel (*p* = 0.0022). NC, normal control; DSS, DSS-induced colitis; NC MLB, mesenteric lymphatic B cells derived from normal control rat; DSS MLB, mesenteric lymphatic B cells derived from DSS-induced colitis rat; NC MLB-DSS, the DSS-induced colitis rats receiving NC MLB; DSS MLB-DSS, the DSS-induced colitis rats receiving DSS MLB. *n* = 6; Mann–Whitney test; * *p* < 0.05; ** *p* < 0.01 with NC MLB-DSS group.

To assess the effects of transferred MLB cells in DSS-induced colitis rats, the phenotype of colitis rats was compared between the two groups. Compared to rats receiving MLB cells derived from the NC group (NC MLB), colitis rats receiving MLB cells from the DSS group (DSS MLB) had a shorter colon length (Figure 5A), greater weight loss (Figure 5B), a higher disease activity index (Figure 5C), and a higher histopathological score (Figure 5D). These findings indicate that MLB cells aggravated DSS-induced colitis.

### 3.4. MLB Cells Aggravating DSS-Induced Colitis Might Be Related to the Alteration of Colonic T Cells

**Figure 6:**
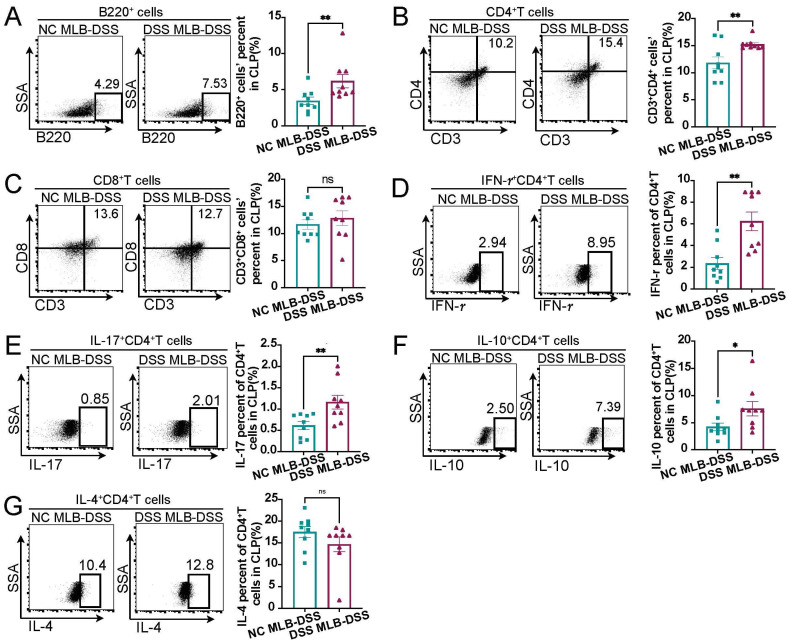
Changes in colonic lamina propria adaptive immune cells in DSS-induced colitis rats when receiving MLB cells isolated from NC and DSS rats. (**A**–**G**) Representative flow cytometry images of adaptive immune cells in colonic lamina propria (CLP) of NC MLB-DSS or DSS MLB-DSS group are shown in the left panel, and statistical analysis between the two groups is displayed in the right panel. B cells, A, *p* = 0.0026; CD4^+^T cells, B, *p* = 0.0292; CD8^+^T cells, C, *p* = 0.4999; IFN-*γ*^+^CD4^+^ T cells, D, *p* = 0.0027; IL-17^+^CD4^+^ T cells, E, *p* = 0.0052; IL-10^+^CD4^+^ T cells, F, *p* = 0.0241; IL-4^+^CD4^+^ T cells, G, *p* = 0.1284; NC MLB, mesenteric lymphatic B cells derived from normal control rat; DSS MLB, mesenteric lymphatic B cells derived from DSS-induced colitis rat; NC MLB-DSS, the DSS-induced colitis rats receiving NC MLB; DSS MLB-DSS, the DSS-induced colitis rats receiving DSS MLB; *n* = 9; Mann–Whitney test; * *p* < 0.05; ** *p* < 0.01 with the NC MLB-DSS group; ns, no significant difference.

Compared to colitis rats receiving NC MLB, rats receiving DSS MLB showed a higher proportion of B220^+^ B cells (Figure 6A) and CD3^+^CD4^+^ T cells (Figure 6B) in the colon, but the treatment had no effect on the proportion of CD3^+^CD8^+^ T cells (Figure 6C). In addition, there was an increased proportion of IFN-γ^+^CD4^+^ T (Th1) cells (Figure 6D), IL-17^+^CD4^+^ T (Th17) cells (Figure 6E), and IL-10^+^CD4^+^ T cells (Figure 6F) in rats receiving MLB cells isolated from DSS rats, with no significant difference in the proportion of IL-4^+^CD4^+^ T (Th2) cells (Figure 6G) between the two groups. These findings demonstrate that MLB cells derived from DSS-induced colitis rats could migrate to the colon and affect the proportion of T cells to aggravate colitis.

### 3.5. MLB Cells Might Migrate to the Colon via CXCR5-CXCL13 in DSS-Induced Colitis

**Figure 7:**
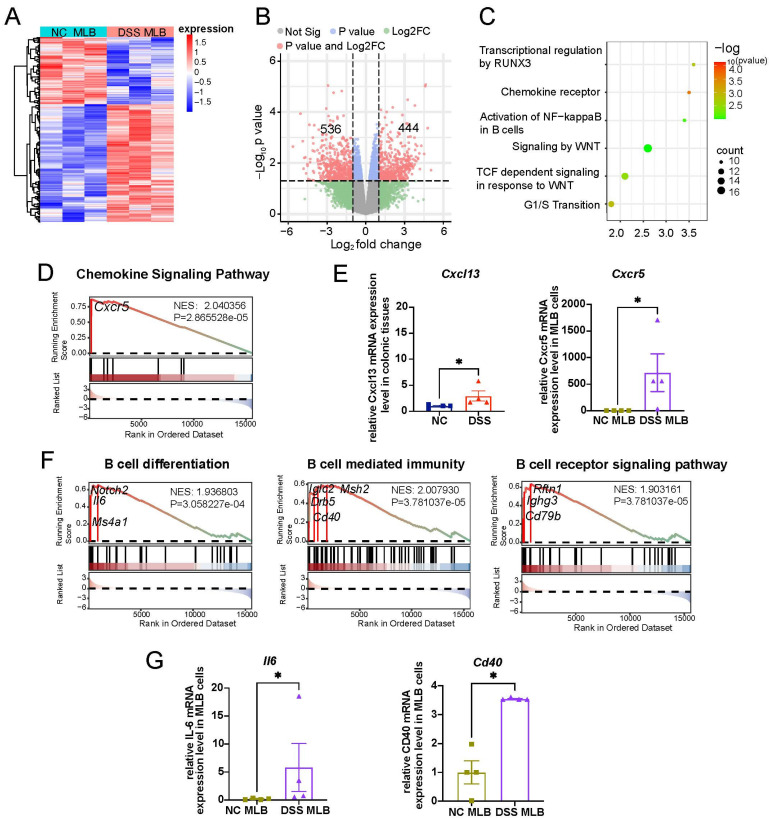
The possible underlying pathways of MLB cell migration to the colon in DSS-induced colitis rats. (**A**) Heatmap displaying differentially expressed genes between NC MLB and DSS MLB. (**B**) Volcano plot analysis illustrates the differentially expressed genes between DSS MLB and NC MLB. (**C**) KEGG analysis demonstrates the enrichment pathways of differentially expressed genes between DSS MLB and NC MLB. (**D**) Enrichment plot and key upregulated genes in B cell-related chemokine signaling pathway between DSS MLB and NC MLB. (**E**) Comparison of *Cxcl13* mRNA expression in colonic tissues and *Cxcr5* mRNA expression in MLB cells between NC and DSS rats using real-time PCR (*Cxcl13*, *p* = 0.0386; *Cxcr5*, *p* = 0.04). (**F**) Enrichment plot and key upregulated genes in B cell differentiation, B cell-mediated immunity, and B cell receptor signaling pathway between DSS MLB and NC MLB. (**G**) Comparison of *Il6* (B cell differentiation) expression and *Cd40* (B cell-mediated immunity) expression between NC MLB and DSS MLB using real-time PCR. NC MLB, mesenteric lymphatic B cells derived from normal control rat; DSS MLB, mesenteric lymphatic B cells derived from DSS-induced colitis rat; NC, normal control; DSS, DSS-induced colitis; *n* = 3–4; Mann–Whitney test; * *p* < 0.05 with NC MLB.

To further explore the potential chemotactic mechanism of MLB cells in the colon of colitis rats, we conducted transcriptome sequencing of MLB cells from both NC and DSS-induced colitis rats. The heat map and volcano plot show 980 significantly altered transcripts (444 upregulated and 536 downregulated) in MLB cells derived from DSS-induced colitis rats (Figure 7A,B). Subsequently, we performed Kyoto Encyclopedia of Genes and Genomes (KEGG) functional analysis and found that the differentially expressed genes were significantly enriched in chemokine receptors, Nuclear Factor Kappa B, Wingless-type pathway, and so on (Figure 7C). Further Gene Set Enrichment Analysis (GSEA) indicated that Cxcr5 obtained the highest enrichment scores in the chemokine signaling pathway, when comparing DSS MLB with NC MLB (Figure 7D). To ascertain whether CXCR5 plays a crucial role in MLB cells migration to the colon, we compared the mRNA expression of Cxcl13 (the specific ligand of Cxcr5) in colonic tissues of NC and DSS-induced colitis rats through real-time PCR. We found that the mRNA expression of Cxcl13 was significantly increased in the colonic tissues of DSS-induced colitis rats (Figure 7E). Additionally, the mRNA expression level of Cxcr5 was significantly elevated in MLB cells derived from DSS-induced colitis rats (Figure 7E). Furthermore, we identified the significant enrichment of genes related to B cell differentiation (Il6, Notch2, and Ms4a1), B cell-mediated immunity (Cd40), and the B cell receptor signaling pathway (Cd79b and Ighg3) in MLB cells derived from DSS rats (Figure 7F). Similarly, we verified the mRNA expression levels of Cd40 and Il6 in mesenteric lymphatic B cells, which are associated with B cell activation and immunoregulation. We found a significant increase in their mRNA expression levels in MLB cells derived from DSS-induced colitis rats by real-time PCR, consistent with the transcriptome results (Figure 7G). Therefore, we speculate that MLB cells might migrate to the colon via the CXCL13–CXCR5 axis in DSS rats.

**Figure 8:**
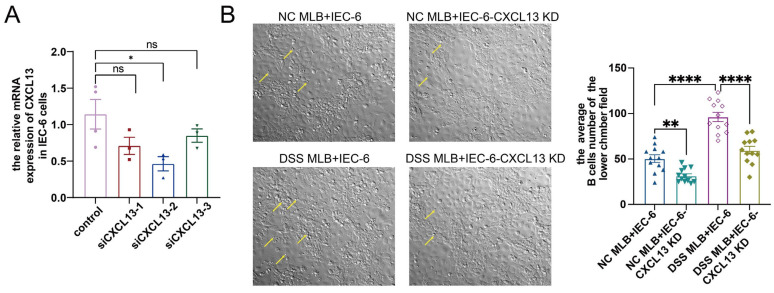
The impact of IEC-6 cell CXCL13-knockdown on MLB cell migration. (**A**) The relative mRNA expression of *Cxcl13* in the treatment of siCXCL13-1 (*p* = 0.1143), siCXCL13-2 (*p* = 0.0286) or siCXCL13-3 (*p* = 0.2), respectively. (**B**) Representative images of B cells (indicated by yellow arrows) in the lower chamber in NC MLB+IEC-6 group, NC MLB+IEC-6-CXCL13 KD group, DSS MLB+IEC-6 group and DSS MLB+IEC-6-CXCL13 KD group are shown in the left panel, and statistical analysis between the four groups is displayed in the right panel. NC MLB+IEC-6, mesenteric lymphatic B cells derived from normal control rat co-culture with IEC-6 cells; NC MLB+IEC-6-CXCL13 KD, mesenteric lymphatic B cells derived from normal control rat co-culture with IEC-6-knocked-down CXCL13 cells; DSS MLB+IEC-6, mesenteric lymphatic B cells derived from DSS-induced colitis rat co-culture with IEC-6 cells; DSS MLB+IEC-6-CXCL13 KD, mesenteric lymphatic B cells derived from DSS-induced colitis rat co-culture with IEC-6-knocked-down CXCL13 cells. NC MLB+IEC-6 group vs. DSS MLB+IEC-6 group, *p* < 0.0001; NC MLB+IEC-6 group vs. NC MLB+IEC-6-CXCL13 KD group, *p* = 0.002; DSS MLB+IEC-6 group vs. DSS MLB+IEC-6-CXCL13 KD group, *p* < 0.0001; *n* = 3–4, Mann–Whitney test for Figure 8A; *n* = 12, one-way ANOVA analysis for Figure 8B; * *p* < 0.05, ** *p* < 0.01, **** *p* < 0.0001, ns, no significant difference.

To further identify the role of CXCR5–CXCL13 in the MLB cells migration, we knocked-down *Cxcl13* mRNA expression using siRNA in IEC-6 cells. Knockdown with siCXCL13-2 decreased the CXCL13 mRNA expression in IEC-6 cells by nearly 50% (Figure 8A), and it was used for the subsequent experiment. IEC-6 cells were treated with either control or siCXCL13-2 for 24 h. Subsequently, the sorted mesenteric lymphatic B cells from normal control rats and rats with DSS-induced colitis were inoculated into the upper Transwell chamber. After co-culture for 24 h, the number of B cells migrating to the lower chamber in the DSS MLB+IEC-6 group was significantly higher than in the NC MLB+IEC-6 group (Figure 8B). Additionally, after CXCL13 knockdown, both numbers of B cells derived from NC MLB or DSS MLB were significantly decreased compared to the indicted control (Figure 8B). The changes in B cell migration to the lower chamber during co-culture were illustrated in Video S1–S4. These findings indicate that CXCL13 plays a crucial role in MLB cells migration and further support the notion that MLB cells migrate to the colon via the CXCR5–CXCL13 axis.

## 4. Discussion

**Figure 9:**
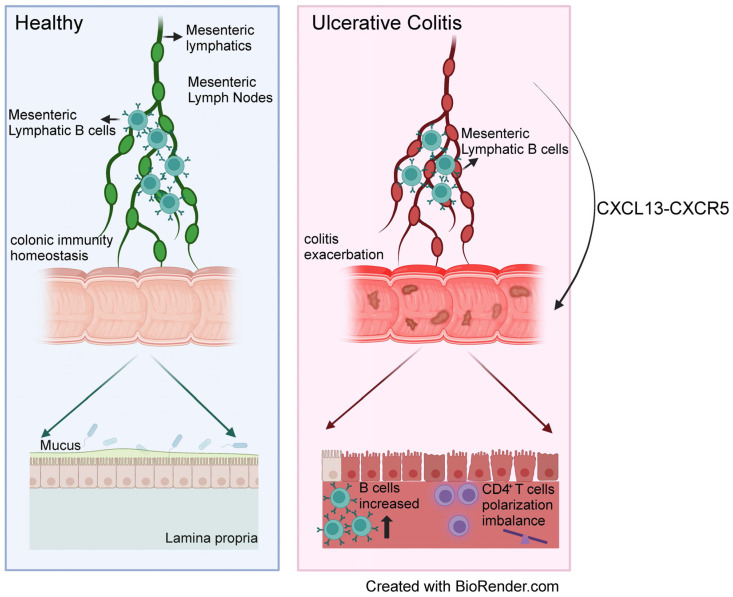
Scheme of mesenteric lymphatic B cells aggravating colitis. The potential mechanism of mesenteric lymphatic B cells (MLB) aggravating colitis may be as follows: MLB cells increase in colitis rats, migration to the gut via the CXCR5–CXCL13 axis, and aggravation of colitis through regulating the immune response of colonic T cells. MLB cells, mesenteric lymphatic B cells.

Inflamed and dysfunctional lymphatics are a hallmark of IBD. Lymphatic B cells participate in lymphatic remodeling and influence the immune response through the regulation of DC migration. These observations lead to the hypothesis that MLB cells may play significant roles in IBD. In this study, we observed an increase in the proportion of MLB cells and the expansion of mesenteric lymphatics in colitis rats. MLB cells derived from DSS-induced colitis rats could migrate to the colon and aggravate colitis, and intestinal T cells responded to the adaptive transfer of MLB cells derived from colitis rats. Further studies indicated that MLB cells migrated to the intestine via the CXCR5–CXCL13 axis (Figure 9). 

The mesenteric lymphatic compartments function under the stimulation of potential exposure and are involved in immune defense. Lymph flow is critical for antigen and DC transport. Lymphatic obstruction or impaired contraction can lead to immunosuppression, edema, hypoproteinemia, and lymphocytopenia [18,19]. In colitis patients and animals, lymphangiogenesis, lymphatic dilation, and a reduction in lymphatic drainage are often observed [5,6,7]. This lymphatic alteration may occur earlier than any cellular inflammatory infiltration or mucosal damage in IBD [20] and can persist even after the removal of DSS [21]. Blocking lymphangiogenesis through vascular growth factor receptor 3-blocking antibodies or angiopoietin 2 deficiency has been shown to aggravate intestinal inflammation in colitis mice [22]. Additionally, the induction of prolymphangiogenic factor [VEGF-C] ameliorated experimental IBD in mice [23]. Furthermore, Yi Yin et al. have demonstrated that lymphatics-targeting drugs suppressed lymphangitis and promoted lymphatic drainage, resulting in improved intestinal inflammation in an experimental colitis model [11]. In our study, we also observed alterations of the mesenteric lymphatic structure in colitis rats. 

It has been demonstrated that B cells are involved in IBD [24], although previous studies targeting B cells indiscriminately proved unsuccessful [25] or even detrimental [26]. B cells exhibit diverse characteristics in various lymphatic tissues. There has been limited research focusing on the role of lymphatic B cells in IBD. Lymphatic B cells drain to the MLN, and many studies have explored B cells in MLN due to the convenience of sample collection. A 4.3-fold expansion in MLN B cell numbers was observed in colitis mice, and the co-transfer of MLN B cells along with CD4^+^ T cells increased colitis compared to the transfer of CD4^+^ T cells alone [27]. The function of B cells in draining the MLN of the inflamed gut was found to be altered in IBD [28]. Transferred MLN CD19^+^IgM^hi^ B cells—but not marginal zone B cells—protected mice from T cell-induced colitis [29]. In CD patients, B cells were found to be aggregated, resembling tertiary lymphoid organs, around mesenteric lymphatics entering and exiting the MLN [13]. However, direct evidence of mesenteric lymphatic B cells participating in IBD is lacking. In this study, we demonstrated that the infusion of mesenteric lymphatic B cells into recipient rats aggravated colitis.

The roles of T cells in IBD have been explored more extensively than those of B cells. Belonging to the adaptive immune system, B cells play a central role in humoral immunity and participate in cellular immunity through interactions with CD4^+^ T cells. In IBD, activated B cells could exacerbate proinflammatory cytokine production by intestinal CD4^+^ T cells through the co-stimulating factor CD40L-CD86, and depletion of B cells diminished intestinal inflammation due to reduced CD4^+^ T cell proinflammatory cytokine production [30]. In the present study, we also found that the proportion of colonic T cells was altered with aggravated colitis induced through MLB adoptive transfer.

We observed higher expression of *Cxcr5* in mesenteric lymphatic B cells and higher expression of *Cxcl13* in the intestine of colitis mice. It is known that all leukocytes can enter the draining lymph nodes, but only lymphocytes can leave the nodes. B cells were found to exit in lymph to the MLN from intestinal mucosa at a speed of six orders of magnitude per hour in mini pigs [31], where they received antigenic stimulation from intestinal DCs. B cells in MLN—but not in Peyer’s patch or peripheral lymph nodes—could migrate to the intestinal mucosal layer and lamina propria, participating in mucosal immunity there through secreting sIgA [32,33]. Chemokines and ligands play pivotal roles in promoting the migration of leukocytes to areas of inflammation, ultimately resulting in mucosal inflammation. CXCR5 is highly expressed in mature B cells [34]. CXCL13 and CXCR5 are required for B cell homing in secondary lymphoid organs [35]. Most lymph nodes were absent in CXCL13^-/-^ and CXCR5^-/-^ mice, consistent with impaired Peyer’s patches [35]. A higher level of serum CXCL13 was correlated with inflammatory responses in IBD patients (including UC and CD) and DSS-induced mice, and colitis was alleviated in CXCL13-deficient mice or those administrated anti-CXCL13 antibodies [36]. The regulatory B cells in the colon increased as colitis was alleviated in CXCL13-deficient mice [36]. Furthermore, the roles of integrin α4β7 and CCR10–CCL28 in inducing the migration of B cells to the colon have also been demonstrated [4].

In this study, we did not distinguish the B cell subtype when exploring their role in DSS-induced colitis. In fact, IL-10-producing B (Breg) cells are thought to play a defense role, while B2 cells are considered to be proinflammatory cells in the immune response. Additionally, to verify the role of MLB cells in colitis, B cells were infused through the tail vein, rather than the mesenteric lymphatic, which did not fully replicate the real path of MLB cells. Due to the limitations of current techniques, leveraging newly developed lymphatic imaging technologies will likely lead to further progress in understanding the role of lymphatics in IBD. Furthermore, novel treatments for IBD might be discovered through modulating MLB cells in the future.

## 5. Conclusions

It was found that MLB cells derived from DSS-induced colitis rats could migrate to the colon and aggravate colitis, where intestinal T cells responded to the received MLB cells derived from colitis rats. Further studies indicated that MLB cells might migrate to the intestine via the CXCR5-CXCL13 axis. 

## Data Availability

The original contributions presented in the study are included in the article. Further inquiries can be directed to the corresponding authors.

## Supplementary Materials

The following supporting information can be downloaded at: https://www.mdpi.com/article/10.3390/biology13050322/s1, Figure S1: The gating strategy used to identify the B220^+^ cells and IL-10^+^ CD4^+^ T cells in Figure 2 and Figure 6. Figure S2: The gating strategy used to identify the CD3^+^ CD4^+^ cells, CD3^+^ CD8^+^ cells, IFN-*γ*^+^ CD4^+^T cells, IL-4^+^ CD4^+^T cells, and IL-17^+^ CD4^+^ T cells in Figure 2 and Figure 6. Figure S3: The positive/negative cut-off definition of cytokines in Figure 2. Figure S4: The positive/negative cut-off definition of cytokines in Figure 6. Video S1: The live cell tracking images of NC MLB co-culture with IEC-6 cells. Video S2: The live cell tracking images of NC MLB co-culture with IEC-6-knocked-down CXCL13 cells. Video S3: The live cell tracking images of DSS MLB co-culture with IEC-6 cells. Video S4: The live cell tracking images of DSS MLB co-culture with IEC-6-knocked-down CXCL13 cells.
