# Supplementary material for: Mesenteric Lymphatic B Cells Migrate to the Intestine and Aggravate DSS-Induced Colitis via the CXCR5–CXCL13 Axis
Source: Biology (Basel). 2024 May 6;13(5):322. doi: 10.3390/biology13050322 (PMC11117591; doi:10.3390/biology13050322)
Supplement: Supplementary file 1 [file biology-13-00322-s001.zip › biology-2963277-supplementary.pdf]

Mesenteric Lymphatic B cells migrate to the intestine and aggravate DSS-induced colitis via CXCR5–CXCL13 axis

Yu Zhang, Zhe Wu, Qinghe Zhao, Yaming Liu, Qing Huang, Menglei Zhang, Shuolei Li, Di Wang, Na Li, Yujing Chi, Yulan Liu

Contents:

Supplementary figures and figure legends

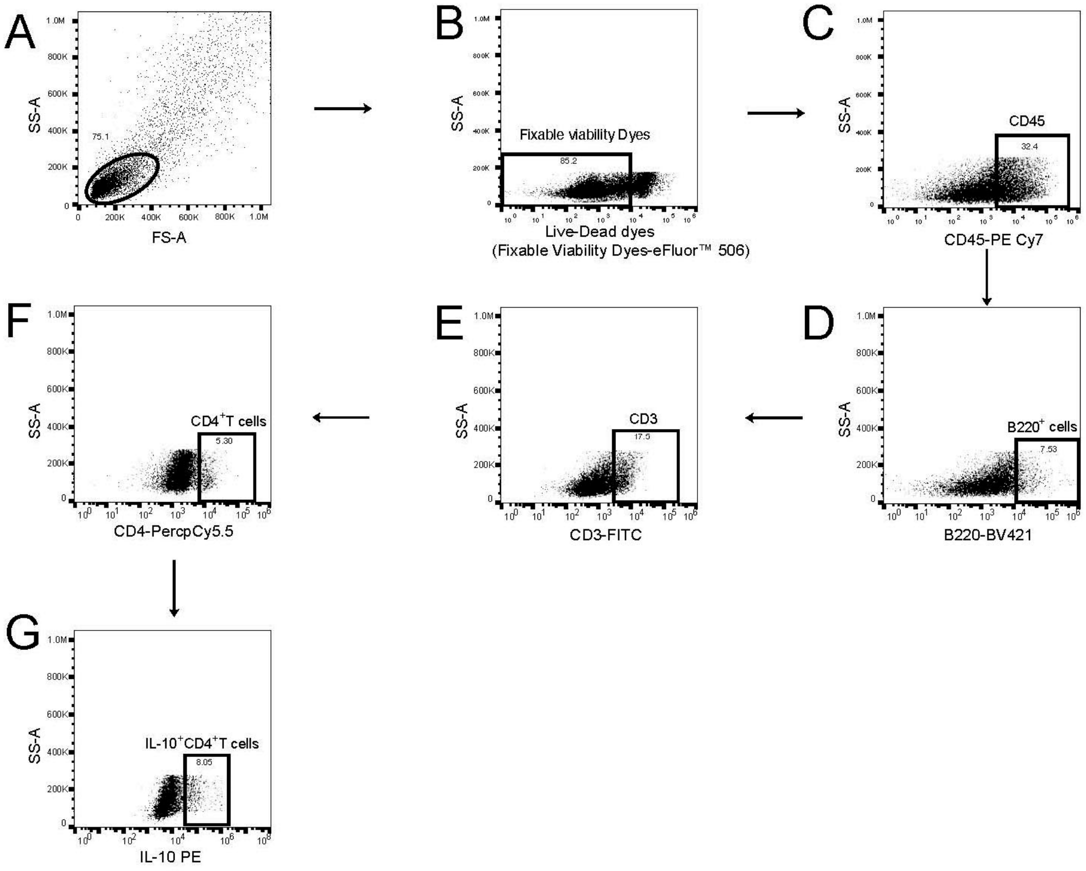

Supplementary Figure S1. The gating strategy used to identify the B220+ cells and IL-10+CD4+ T cells in Figure 2 and Figure 6.

Gates are shown sequentially from A to G according to the arrows indicate.

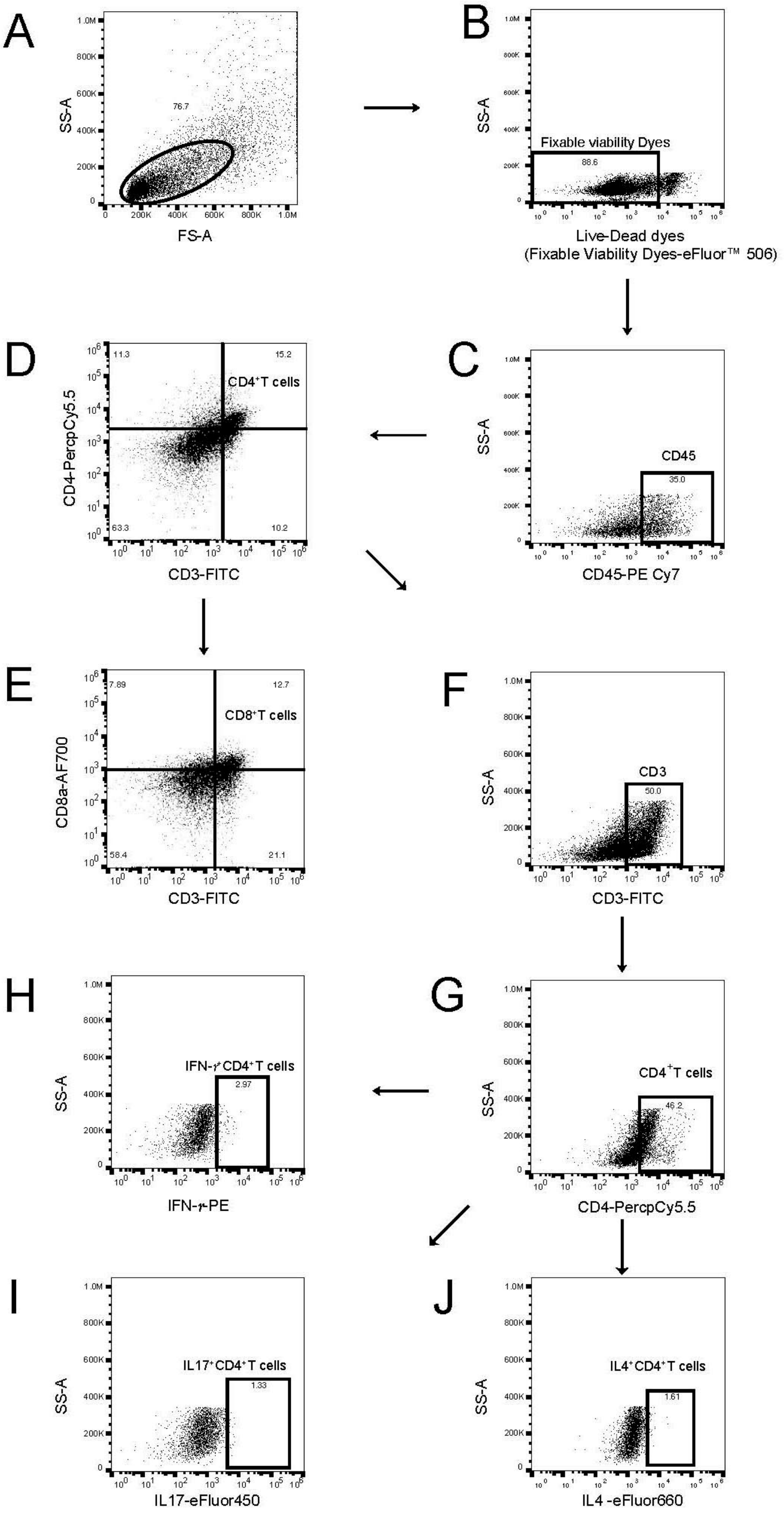

Supplementary Figure S2. The gating strategy used to identify the CD3^{+}CD4^{+} cells, CD3^{+}CD8^{+} cells, IFN-γ^{+}CD4^{+}T cells, IL-4^{+}CD4^{+}T cells, and IL-17^{+}CD4^{+} T cells in Figure 2 and Figure 6.

Gates are shown sequentially from A to J according to the arrows indicate.

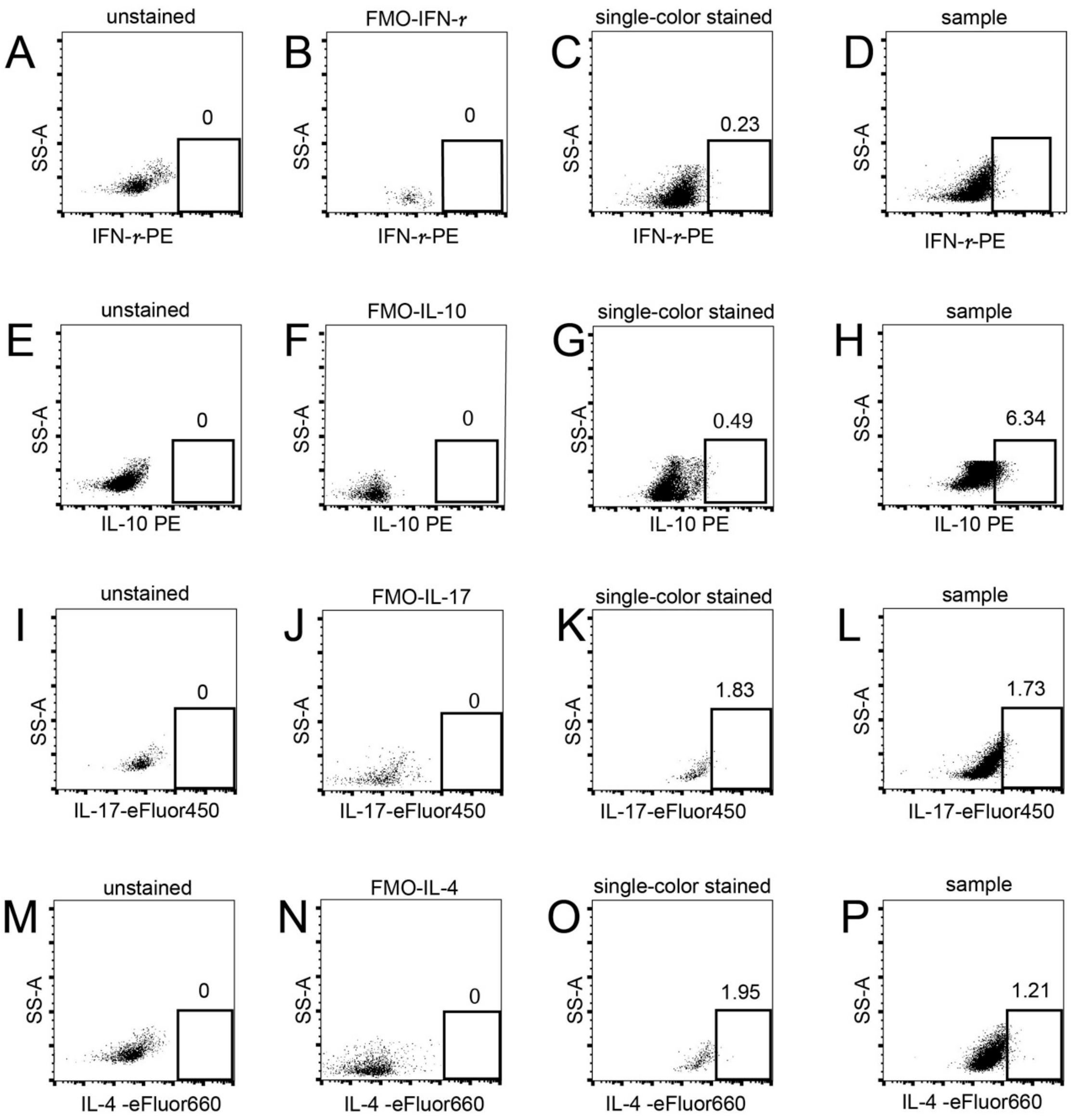

Supplementary Figure S3. The positive/negative cut-off definition of cytokines in Figure 2.

A-D. The unstained (A), FMO-IFN-γ (B), single-color stained (C) and sample tubes (D) of IFN-γ in Figure 2. E-H. The unstained (E), FMO-IL-10 (F), single-color stained (G), and sample tubes (H) of IL-10 in Figure 2. I-L. The unstained (I), FMO-IL-17(J), single-color stained (K), and sample tubes (L) of IL-17 in Figure 2. M-P. The unstained (M), FMO-IL-4 (N), single-color stained (O), and sample tubes (P) of IL-4 in Figure 2.

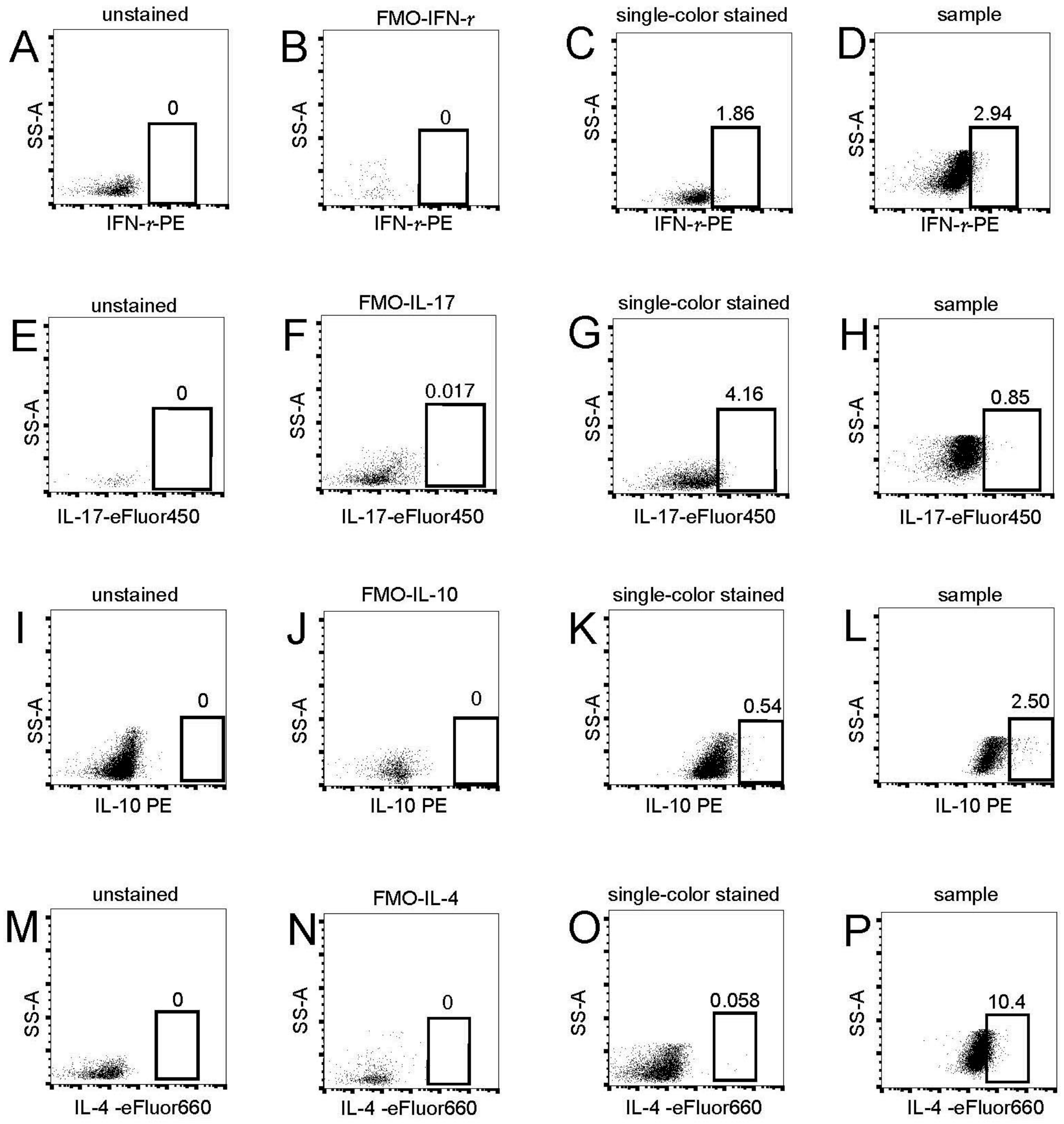

Supplementary Figure S4. The positive/negative cut-off definition of cytokines in Figure 6.

A-D. The unstained (A), FMO-IFN-γ (B), single-color stained (C) and sample tubes (D) of IFN-γ in Figure 6. E-H. The unstained (E), FMO-IL-17 (F), single-color stained (G), and sample tubes (H) of IL-17 in Figure 6. I-L. The unstained (I), FMO-IL-10(J), single-color stained (K), and sample tubes (L) of IL-10 in Figure 6. M-P. The unstained (M), FMO-IL-4 (N), single-color stained (O), and sample tubes (P) of IL-4 in Figure 6.
